# Predicting mass transfer activation energy and physicochemical properties of dried onion using numerical modeling and artificial intelligence

**DOI:** 10.1038/s41598-025-04814-7

**Published:** 2025-06-03

**Authors:** Fangfang Liu, Hany S. El-Mesery, Ahmed H. ElMesiry, Zicheng Hu, Ali Salem

**Affiliations:** 1 School of Mechanical and Electrical Engineering, Suqian University, Suqian, 223800 China; 2 School of Energy and Power Engineering, Jiangsu University, Zhenjiang, 212013 China; 3 Agricultural Engineering Research Institute, Agricultural Research Center, Dokki, Giza, 12611 Egypt; 4 Faculty of Computer Science and Engineering, New Mansoura University, Mansoura, 35742 Egypt; 5 Civil Engineering Department, Faculty of Engineering, Minia University, Minia, Egypt; 6 Structural Diagnostics and Analysis Research Group, Faculty of Engineering and Information Technology, University of Pécs, Pécs, 7622 Hungary

**Keywords:** Artificial intelligence, Onion, Thin-layer equations, Quality, Computational simulation, Biophysical chemistry, Agroecology, Plant sciences

## Abstract

The quality of the onion slices was statistically evaluated based on the variables of drying conditions, considering the following characteristics: drying time, color, shrinkage, water activity, and rehydration ratio, critical parameters in evaluating food processing and preservation methods. The onion slices were subjected to drying under the ensuing settings: infrared powers of 1500, 3500, and 5500 W/m^2^; airflow rates of 0.3, 0.7, and 1.0 m/s; and air temperatures of 40, 50, and 60 °C. The findings indicated that the drying time increased with air velocity to 0.7 and 1.0 m/s at a constant infrared intensity of 5500 W/m^2^, resulting in a significant increase of 26 and 37%. The water activity values for the sample ranged from 0.40 to 0.49. The maximum vitamin C content was 24 mg/g, followed by an air temperature of 40 °C and 0.3 m/s air velocity under 1500 W/m^2^. Conditions of 1.5 m/s, 40 °C, and 1500 W/m^2^ indicate the lowest rehydration ratio. The ANN model proved to be a robust tool for predicting and optimizing drying parameters, including drying duration, energy consumption, and quality. Additionally, it highlights the potential of advanced heating technologies to optimize food drying approaches, providing important insights for the food sector in its quest for increased efficiency.

## Introduction

Due to their high moisture content, agricultural products such as vegetables and fruits are classified as perishable. Free water elimination is an effective method for preserving food for off-season usage, as it prevents enzymatic reactions and microbiological deterioration^1^. Dehydrating is a commonly known procedure that decreases the requirement for storage intergalactic and transport mass, prevents the product from absorbing free water, and extends its shelf life. Dried fruits are optimal for making fruits more available, as they have an extended shelf life. It is also crucial to progress and increase the marketplace for best, consumer-dried foods with satisfactory color, form, and rehydration abilities^2^.

The application of infrared technology for drying agricultural products is becoming increasingly prevalent, with traditional methods being supplanted in many instances. The utilization of the infrared drying method in foods has numerous benefits. The direct heating of the material facilitates the rapid drying process. Furthermore, infrared drying requires significantly less energy than hot-air drying. Infrared energy is a form of electromagnetic radiation located just beyond the red end of the visible light spectrum. It is frequently utilized for heating due to its capacity to be absorbed by the substance and transformed into thermal energy^3^. A heating element engineered to facilitate infrared energy transfer is intended to emit infrared radiation. This can be accomplished using many methods, including carbon or ceramic components, which release infrared radiation upon heating. Rather than warming the surrounding air, these heating components convey heat directly to the material’s surface via infrared radiation. This method enhances efficiency by circumventing the necessity to heat air, thereby allowing heat transmission to the substance through convection^4^. This heating technology may improve energy efficiency by reducing heat loss to the ambient air. Heat is precisely directed to the required areas, resulting in expedited heating times and less energy use. This heating element is utilized in diverse applications, such as infrared heaters in home and commercial environments, industrial procedures necessitating direct material heating, and certain cooktops and ovens. The unheated surrounding air enhances safety and comfort in the environment. In a space heated by infrared radiation, the air temperature may be lower than conventional heating; nonetheless, objects and individuals within the room might still experience warmth due to direct heat transfer^5^.

Several empirical and semi-empirical mathematical models have been utilized to analyze food drying. At the same time as it may be used to recreate the drying curve under identical conditions, the thin-layer model can also be used to estimate the mass transfer that occurs throughout the drying process^6^. The mathematical modeling represents the optimal methodology for characterizing the kinetics of the drying procedure. The conceptualization and modeling of various mass movement operations, such as a process known as dehydration and the emission of water during preservation, can be enhanced by appropriate water diffusivity^7^.

Modeling complicated systems and solving nonlinear issues, especially with inadequate or unclear data, are common uses of artificial neural networks (ANNs). With the help of ANNs and the underlying relationships in the data, it is possible to process numerous inputs and generate multiple outputs^8^. Machine learning in food processing has been heavily applied over the past decade, especially in modelling and drying process optimization. Various machine-learning models, including ANN, SVM, and RF, have been utilized to predict drying times, minimize energy consumption, and assess final product quality^9^. Machine learning has also been explored for real-time monitoring and control of food processing to enhance efficiency and quality. While these recent advances have been in machine learning, many studies rely on a very limited dataset and simple models, which cannot represent the fundamental interactions occurring in drying. The use of ANNs to model drying processes has been fruitful for researchers. For example^10^, used various ANN models to forecast how rough rice dried with hot air and infrared drying, including changes in moisture content, drying time, failure force, and broken kernel %. They compared various training techniques, transfer functions, and network topologies to refine the prediction process. Other research has also shown that ANNs may accurately predict drying properties^11^. Applications of real-time monitoring and adaptive control, enabled by machine learning, during infrared (IR) drying processes have been limited. Real-time adjustments based on ML algorithms could substantially benefit energy efficiency and product quality. Therefore, machine learning can better capture these complex interactions and give more precise predictions concerning drying kinetics and product quality than traditional approaches^12^. Jafari et al.^13^ found that the ANN model outperformed mathematical modeling methods regarding productivity and accuracy when predicting variations in the moisture ratio of green bell peppers during hot air fluidized bed drying. Similarly, El-Mesery et al.^14^ applied an ANN to predict drying kinetics and the quality attributes of garlic slices during continuous infrared-assisted hot air drying. Zalpouri et al.^15^ investigated the onion puree of varying thicknesses. They were dried using RWD and CD methods to evaluate drying kinetics, physicochemical qualities, and thermal analysis of the dried powder. The results showed an increase in the thickness of the puree from 2 to 6 mm, and drying time increased from 135 to 240 min for RWD and 510 to 660 min for CD. MLF-ANN, with a back-propagation algorithm, was used to predict the accurate MR of onion puree using RWD and CD. ANN was an effective method to predict MR for both drying methods. There has been a lot of focus on applying ANNs to anticipate drying parameters, but energy performance forecasting throughout drying has received less attention. Not to mention that most of the studies on hybrid infrared drying of agricultural items have taken place in labs rather than outdoors, where the actual drying process can be more unpredictable. Despite the extensive research in this area, much is still to be learned about drying food products using infrared energy. For instance, no study examined the impact of sample thickness and drying conditions on the quality of onion slices in an infrared heating system. The objective of this study was to evaluate the impacts of airflow, slice thickness, and infrared power on drying time, as well as physics-quality strictures and drying kinetics.

## Materials and methodology

### Sample preparation

Fresh Bijapur white onions (*Allium cepa L*.) were used in the present study because they had high solid content (17% w/w), high productivity, and high pungency. Most commercial onion dehydrators also use this variety. The white onions were acquired from a market farm and refrigerated at 4 °C and 70% RH. Onions were taken from storage and allowed to equilibrate with ambient conditions for about 2 h, followed by hand peeling. The peeled onions were cut into circular slices of thickness equal to 4 ± 0.12 mm using a stainless steel cutter.

### Experimental setup

The apparatus incorporates a conveyor belt system, a drying cabinet, an infrared technique, and a convective technique with associated measurement capabilities. The drying space comprises chambers with dimensions (0.8 × 0.8 × 0.60 m). An aluminum layer with an infrared reflective coating measuring 0.15 cm in thickness was applied to the inner surface of the drying chamber. A layer of asbestos, with a thickness of 5 cm, was utilized as an insulating material for the external walls. Within the drying chamber, infrared heater halogen lamps with a wattage of 1000 W and a diameter of 35.5 cm were installed. The lamps also had a diameter of 0.6 cm. The infrared lamps with tray were located in parallel with a stable 15 cm slit. The output power of the infrared radiation can be modified by regulating the voltage with the aid of a power regulator. The convective unit comprises two electrical heaters and a 1.4 kW fan, responsible for generating the requisite drying airflow. Subsequently, the air flows through a PVC tube and into the drying cabinet via two inlets. A control valve was installed at the PVC pipe’s intake to regulate the air flow entering the drying chamber. As the air passed through a pair of electric heaters with a power output of 1.5 kW, its temperature increased. A temperature control apparatus was employed to autonomously activate and deactivate each infrared heater by the prescribed temperature provisions. The airflow rate was adjusted via a manually operated valve with an air speed meter. The anemometer exhibited an employed variety of 0.2 to 20 m/s and demonstrated a precision of ± 0.1 m/s in determining airflow. T-type thermocouples were employed to measure the hot air temperature, with the data recorded by a data recorder with a precision of ± 11 °C.

### Drying operation

The appliance was deactivated for thirty minutes to maintain uniform drying settings. A thin layer of 500 g of onions was evenly spread on the dryer beneath the wire mesh tray. The onion dehydrating employed the following variables: air temperature of 40, 50, and 60 °C; airflow rates of 0.3, 0.7, and 1.0 m/s; and infrared intensity of 1500, 3500, and 5500 W/m². The selection of drying conditions (infrared radiation intensity, air temperature, and air velocity) was based on a literature review, preliminary experiments, and practical considerations for optimizing drying efficiency and quality retention in onion slices. The intensity range was selected based on existing studies on food drying. It ensures the effective penetration of infrared waves into onion slices while avoiding excessive thermal degradation. Prior research has shown that intensities below 1500 W/m² lead to prolonged drying times, while intensities beyond 5500 W/m² can cause excessive heating, leading to undesirable color changes and nutrient loss. This range is consistent with recommendations in the literature, such as El-Mesery and Mwithiga^16^ who suggest that the infrared radiation intensity ranges from 0.15 to 0.30 W/cm^2^. While infrared drying primarily relies on radiation, airflow remains critical in removing evaporated moisture. If airflow is too low, moisture stagnation occurs, resulting in a deceleration of drying time. Conversely, excessive airflow can cool the surface, reducing IR efficiency. Airflow velocities were selected to investigate their impact on moisture removal and energy efficiency. This range encompasses values commonly used in convective drying studies, such as Qenawy et al.^17^ who employed 0.3–1.5 m/s velocities. Although infrared radiation directly affects the material, air temperature influences the convective heat transfer component, which can enhance or hinder drying. The chosen range reflects conditions commonly used in hybrid infrared-hot air-drying systems, optimizing drying kinetics without causing excessive thermal stress on onion slices. Preliminary tests showed that temperatures below 40 °C resulted in inefficient moisture removal, while temperatures above 60 °C could cause thermal damage, such as browning and texture degradation. This range is consistent with recommendations in the literature, such as Pendre et al.^18^ who suggest temperatures of 50–60 °C for hot air drying of the sample to maintain quality. The drying procedure was reiterated till the onion slices attained a final water content of about 6 ± 0.3% (wb).

### Moisture content determination

Using the method outlined by^19^ a sample of onion slices weighing about 20 grams was dried on a plate in an oven at 105 °C for twenty-four hours. This was done to control the original water content of the onion slices. A more reliable average was obtained by conducting three separate runs to generate the average. The process was repeated three times to provide a more reliable average. The initial moisture content of the samples was determined to be 85.8 ± 0.1% weight-to-volume. The MC of the slices, abbreviated as MCdb, was presented in Eqs. 1^20^:

1 $${\text{M}\text{C}}_{db}= \frac{{\text{W}}_{i}-{\text{W}}_{\text{d}}}{{\text{W}}_{d}}$$

The final moisture content (M_f_) was determined on dry bases following Eq. 2^21^.

2 $${M}_{f}= \frac{{W}_{wet}- {W}_{d}}{{W}_{d}}$$

Equation 3 gives the Mt of the dry sample over time t^22^.

3 $${M}_{t}= \left[\frac{\left({M}_{i}+1\right) {W}_{0}}{{W}_{t}}-1\right]=\frac{{W}_{t}-{W}_{d}}{{W}_{d}}$$

### Moisture effective diffusion

The drying of food products is achieved by interior diffusion, typically during the time-decreasing rate. Fick’s second rule has been the basis for developing several mathematical equations published to depict drying processes throughout the decreasing rate phase. To demonstrate, use Eq. 18^1^.

4 $$\frac{\partial M}{\partial t}={D}_{eff}\frac{{\delta }^{2 }M}{\delta {x}^{2}}$$

Initial conditions (t = 0):

5 $$M={M}_{o} 0\le X<L$$

Boundary conditions (t > 0)

6 $${\left.\frac{\delta M}{\delta X}\right|}_{x=0 }=0$$

7 $$M=0 X=L$$

Equation 8 can be used to present the unstable state diffusion equation for slab geometry^22^.

8 $$MR=\frac{M}{{M}_{i}}= \frac{8}{{\pi }^{2}} \sum _{n=0}^{\infty }\frac{1}{{\left(2n-1\right)}^{2}}\text{exp}\left[-{\left(2n-1\right)}^{2}{ \pi }^{2} \frac{{D}_{eff}}{4{L}^{2}} t\right]$$

Where t is the drying time in a few seconds, D_eff_ in m^2^/s, L is half the sample thickness (m), and MR is the moisture ratio.

9 $$MR=\frac{8}{{\pi }^{2}}\text{exp}\left[-\frac{{\pi }^{2}{D}_{eff} t}{4{L}^{2}}\right]$$

10 $$\text{ln}\left(MR\right)=\text{ln}\left(\frac{8}{{\pi }^{2}}\right)-\left({\pi }^{2}\frac{{D}_{eff}}{4{L}^{2}}t\right)$$

The slopes approach is utilized to calculate the diffusion coefficient. Equation 11 uses the inclination of the graph of ln MR vs. time at different temperatures to determine the Deff.

11 $$Slope=\frac{{\pi }^{2}{D}_{eff}}{{4L}^{2}}$$

### Activation energy

Equation 26 shows how the energy of activation (Ea) was computed using an Arrhenius-type equation^23^.

12 $${D}_{eff}={D}_{o}\text{exp }\left(-\frac{{E}_{a}}{R {T}_{a}}\right)$$

Where Ea is the activation energy (kJ/mol), R is the universal constant of gas (kJ/mol), T is the standard air temperature (K), and D0 is the Arrhenius equation’s pre-exponential factor (m^2^/s). The energy required for activation was estimated by dividing the slope of the Arrhenius plot by 1/T.

13 $$\text{ln}{D}_{eff}=\text{ln}{D}_{o}-\frac{{E}_{a}}{R}\frac{1}{{T}_{a}}$$

A plot of ln Do vs. 1/T from Eq. (13) yields a slope (K) from Eq. 14^24^.

14 $$k=\frac{{E}_{a}}{R}$$

### Water activity

The samples (3 g) were subjected to a water activity (a_w_) assessment at 25 ± 0.1 °C. The apparatus employs a resistive, electrolytic sensor to quantify the moisture content of the surrounding air within a regulated chamber, thereby facilitating an accurate and dependable assessment of the water activity (a_w_)^25^.

### Colour measurement

The material was subjected to 5 colour tests. The fresh item’s total colour alteration and browning index (BI) was calculated utilizing Eqs. 15 and 30^26^:

15 $$\delta E= \sqrt{{\left({L}^{*}-{L}_{o}^{*}\right)}^{2}+{\left({a}^{*}-{a}_{o}^{*}\right)}^{2}+{\left({b}^{*}-{b}_{o}^{*}\right)}^{2}}$$

16 $$BI= 100 \times \left(\frac{x-0.31}{0.17} \right)$$

17 $$X= \frac{{a}^{*}+1.75{ L}^{*}}{\left(6.645 {L}^{*}+{a}^{*}-3.012{ b}^{*}\right)}$$

### Shrinkage ratio

Different dehydration techniques notice nutrition shrinkage as a typical chemical reaction. An accurate evaluation of the water and temperature profiles in hydrated objects is essential, as these variations impact the outcome. Subsequently, the mean principles were reported^27^. The shrinkage ratio (Sr) was determined using Eq. 18.

18 $$\text{S}\text{r}= 1-\frac{{\text{V}}_{\text{d}}}{{\text{W}}_{o}}$$

where V_o_ is the volume of fresh okra, and V_d_ is the volume of dried slices.

### Rehydration ratio

The present study introduced 150 ml of purified water into a 500 ml beaker. Subsequently, the glass was closed and heated for five minutes. After measuring the samples, the rehydration ratio (Rr) was calculated using Eq. 19^28^. This approach is outlined by^29^.

19 $$RR= \frac{{W}_{r}}{{W}_{d}}$$

*W*_*d*_ is the weight of dried slices (g), and *W*_*r*_ is rehydrated slices (g).

### Vitamin C

The 2,6-dichlorophenol indophenol standard titration method was used to determine the sample’s vitamin C content^30^. The rate of vitamin C retention was determined using Eq. 20^31^.

20 $$VC= \frac{{V}_{t}}{{V}_{o}}$$

### Machine learning model development

This study focuses on the applicability of ANNs to predict the performance of a convective-infrared dryer. The ANN model has been developed to predict the quality performance and drying kinetics. Artificial Neural Networks (ANNs) are a powerful, data-driven artificial intelligence technique. An ANN comprises three layers: input, hidden, and output. Data is transmitted via the neurons from an input layer to the layers below. The neurons in the output and input layers significantly impact the variables. Among these structures, the multi-layer feed-forward network is widely applied in modeling agricultural and food systems. The feed-forward neural network has an input layer (n), an output layer (m), and one or more hidden layers (h). The number of neurons in the input and output layers represents the number of independent variables (input) and dependent variables (output), respectively. Each node has a weight connected to all the nodes in the next layer, which is calculated to give the sum of the nodes (x) representing an activation input value function of the node. The value of x is computed first, followed by computing the activation function of the node until the output node’s activation function is acquired. A hidden layer with different nodes is used to process the information received by the input nodes through the activation function. This study used a multilayer feed-forward network structure with three input parameters (air temperature, air velocity, and infrared intensity), 1–3 hidden layers, and one output parameter (water activity, color, shrinkage ratio, rehydration ratio, and vitamin C).

The ANN model was successfully integrated with other methods, mainly the Self-Organizing Map (SOM) approach, to optimize drying conditions and provide valuable data on drying conditions. The SOM procedure, a key example of unverified learning in neural networks, organizes varied data into a 2D feature map. The network architecture: a single layer applies these conditions to the neurons, which then successfully combine to form n × m clusters. Organizing these output parameters into 20 × 20 clusters resulted in 400 output neurons. Clustering was successfully executed using a method comparable to analytical techniques. This approach facilitates the creation of well-organized and understandable data groupings.

The back-propagation technique, utilizing the sigmoid function, is employed during the ANN training stage. For the ANN model, private code was developed using MATLAB software. The dataset was randomly split, with 70% allocated for training and 30% for testing. The ANN setup with 12 neurons across two hidden layers offered the optimal balance between performance and regression accuracy. Back-propagation is a training method for ANN that remains stable at low learning rates. Additionally, all cases are subjected to the sigmoid function, as described in Eqs. 21,^17^:

21 $$f\left(x\right)=\frac{1}{1+{e}^{-x}}$$

Before being integrated into the ANN framework, the data set was adjusted according to Eqs. 22,^32^:

22 $${X}_{i}=\frac{{x}_{i,max}-{x}_{i}}{{x}_{i,max}-{x}_{i,min}}$$

### Computational simulation approach

Numerical modeling of the dehydrating reactions and dynamics is a controlling mechanism for procedures that can be utilized to ascertain the optimal dehydration method for a slice. The developed models facilitate the construction of new dehydrating structures, the identification of optimal drying constraints, and the anticipation of concurrent mass and heat transport phenomena throughout the drying process. Precise modeling of drying behavior leads to producing excellent goods and enhanced energy efficiency. This research employed eleven semi-empirical dehydrating models to delineate the dehydrating kinetics of onions^33^.

23 $$\text{M}\text{R}= \frac{{M}_{t}-{\text{M}}_{\text{e}}}{{\text{M}}_{\text{i}}-{\text{M}}_{\text{e}}}$$

Because the equilibrium moisture content (M_e_) is insignificant, the moisture ratio can be calculated using Eq. 24^34,35^.

24 $$\text{M}\text{R}= \frac{\text{M}}{{\text{M}}_{\text{i}}}$$

Equation 25 was operated to compute the (Dr) drying rate^36^.

25 $$\text{D}\text{r}= \frac{{\text{M}}_{t+dt}-{\text{M}}_{\text{t}}}{\text{d}\text{t}}$$

A non-linear numeric analysis was carried out to incorporate the numerical information into 11 semi-theoretical thin-layer equations (Table 1).

**Table 1:** The mathematical modelling expanded to the drying kinetics.

No.	Models’ name	Models’ equation	Refs.
1	Logarithmic model	MR = a. exp(-kt) + c	^37^
2	Page	MR = exp (-kt^n^)	^38^
3	Newton	MR = exp (-kt)	^39^
4	Midilli and Kucuk	MR = a. exp(-kt^n^) + b.t	^40^
5	Wang and Singh	MR = 1 + at + bt^2^	^41^
6	Verma et al.	MR = a exp(-kt) +(1-a) exp(-gt)	^42^
7	Modified Page	MR = exp [-(kt)^n^]	^43^
8	Modified Henderson and pabis	MR = a exp(-kt) + b exp(-g.t) + c exp(-h.t)	^4^
9	Henderson and Pabis	MR = a. exp(-kt)	^45^
10	Two-term	MR = a exp(-k_0_t) + b exp(-k_1_t)	^46^
11	Thomson	t = a ln (MR) + b [ln (MR)]^2^	^47^

The experimental data are presented as means ± standard deviations (SD). Statistical analyses were performed using SPSS statistics software. The effects of various operating settings on the drying properties and quality parameters were assessed using an analysis of variance (ANOVA), which proceeded by a post hoc Duncan’s multiple range test at a significance rank of 0.05. The equation with the lowest RMSE, χ^2^, and maximum R^2^ values was optimal, as it most clearly defined the drying kinetics^48,49^. The Levenberg-Marquardt algorithm minimizes errors, achieving the minimum error after 1000 iterations. Statistical metrics were utilized to evaluate the model’s performance, including the root mean square error (RMSE) and correlation coefficient (R²)^50^.

26 $${{\upchi }}^{2 }= \frac{\sum _{\text{i}=1}^{\text{N}}{\left({\text{M}\text{R}}_{\text{e}\text{x}\text{p}.\text{i}}-{\text{M}\text{R}}_{\text{p}\text{r}\text{e}.\text{i}}\right)}^{2}}{\text{N}-\text{n}}$$

27 $$RMSE=\sqrt{ \frac{\sum _{i=1}^{N}{\left({MR}_{exp.i}-{MR}_{Pre.i}\right)}^{2}}{N}}$$

28 $${\text{R}}^{2}=\frac{\sum _{\text{i}=1}^{\text{N}}{\left({\text{M}\text{R}}_{\text{e}\text{x}\text{p}.\text{i}}-{\text{M}\text{R}}_{\text{P}\text{r}\text{e}.\text{i}}\right)}^{2}}{\sqrt{\left[\sum _{\text{i}=1}^{\text{N}}{\left({\text{M}\text{R}}_{\text{e}\text{x}\text{p}.\text{i}}-{\text{M}\text{R}}_{\text{P}\text{r}\text{e}.\text{i}}\right)}^{2}\right]\times \left[\sum _{\text{i}=1}^{\text{N}}{\left({\text{M}\text{R}}_{\text{e}\text{x}\text{p}.\text{i}}-{\text{M}\text{R}}_{\text{P}\text{r}\text{e}.\text{i}}\right)}^{2}\right]}}$$

## Results and discussion

### Drying time

**Fig. 1:**
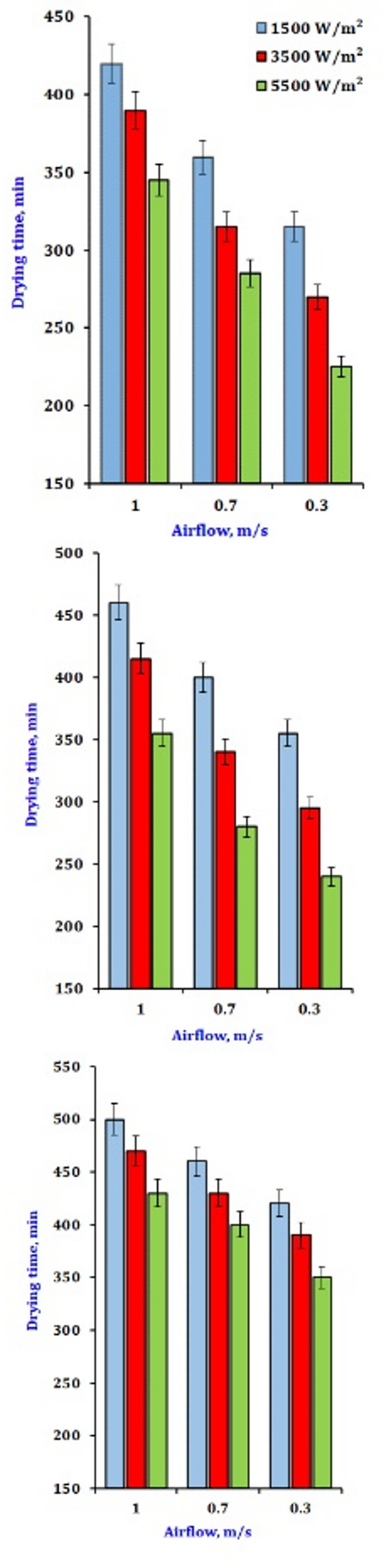
The effect of drying conditions on drying time for dry onion slices.

Figure 1 illustrates the impact of various drying parameters on the duration of the infrared drying. The drying durations required to reduce the water content to about 6% (wb) are shown. Increased infrared power will result in higher product temperatures and heat absorption, enhanced water transmission pushing force, accelerated dehydrating rate, and reduced drying duration. Convection hot air drying is heat transfer between a solid surface and a moving fluid. A drying system usually involves hot air or a hot gas flowing over the wet material. Conversely, forced convection is achieved using a fan or a blower to move the fluid over the material at a higher velocity. Forced convection is more commonly used in industrial drying processes because it provides a higher heat transfer rate and more control over the drying conditions^51^. The heating process is surface-driven since the heat transfer starts at the surface. In thick materials, the temperature gradient from the surface to the interior can be significant, and it may take a relatively long time for the interior to reach a temperature high enough for efficient drying. This can lead to a situation where the surface dries out first, potentially forming a crust that can impede further moisture diffusion from the interior to the surface^52^.

Conversely, the longest drying time occurred under conditions of the highest air velocity combined with the lowest temperature and infrared power, highlighting the combined impact of these factors on drying. The rise in air velocity, while maintaining a constant level, resulted in an extended drying time attributed to the intensity of infrared radiation. The data indicate a decrease in the product’s drying rate with increased air velocity. This pattern was applicable at all levels within the parameters of the current study. Radiation is the transfer of heat in the form of electromagnetic waves. In drying, infrared radiation can supply heat to the wet material. Infrared drying is often used for materials sensitive to high-temperature air or products requiring a quick drying process. For example, in the printing industry, infrared dryers are used to dry inks quickly without affecting the quality of the printed material^53^. Heat is generated within the material as the molecules absorb infrared energy, which can lead to a more rapid increase in temperature in the interior of the material. As a result, the drying process can start from the inside out, reducing the possibility of surface-only drying and crust formation. However, the penetration depth is still limited. In materials with high opacity to infrared radiation, such as some highly pigmented or thick materials, the radiation may only affect the surface layers, and the heat has to be conducted further into the material from the surface, similar to conduction-dominated processes^54^. The item in question will cool, decreasing the dripping airflow. The optimal combination of intensity and airflow rate may reduce drying duration. As shown below in Eq. 29, the drying time could be calculated within the parameters of this work for any recorded air temperature (T °C), airflow (V-m/s), and intensity value (IR-W/m^2^).

29 $$Drying time=616.12-\left(283.7*IR\right)+\left(50.21*V\right)-\left(5.08*T\right)$$

### Computational modeling simulation

The average outcomes of the statistics regarding how much data conforms to various dehydrating models (Tables 2, 3 and 4). All equations accurately predicted drying behavior. This indicates that each model can effectively suggest the infrared dehydrating process of onions. The Midili and Kucuk model demonstrated the most muscular fit, evidenced by the most significant R^2^ values and the lowest χ^2^ and RMSE. After the statistical analysis, the constants from each model were employed to evaluate the MR for each specified value. Additionally, at reduced radiation intensities, the expected sites exhibit a lower degree of distribution. The “Midilli and Kucuk” was modified to accommodate statistics collected at different levels of infrared and airflow, reflecting its relevance.

**Table 2:** The statistical variables were found by fitting numerous thin-layer drying equations at an air temperature of 40 °C.

Models’ name	Air velocity 0.3 m/s								
	1500 W/m^2^			3500 W/m^2^			5500 W/m^2^		
	*R* ^2^	χ^2^	RMSE	*R* ^2^	χ^2^	RMSE	*R* ^2^	χ^2^	RMSE
Logarithmic model	0.905	0.0017	0.010	0.993	0.0012	0.054	0.996	0.0063	0.031
Page	0.991	0.0031	0.052	0.990	0.0032	0.027	0.981	0.0021	0.067
Newton	0.994	0.0051	0.032	0.992	0.0041	0.046	0.995	0.0031	0.076
Wang and Singh	0.993	0.0023	0.052	0.995	0.0030	0.034	0.999	0.0041	0.014
Verma et al.	0.981	0.0061	0.023	0.978	0.0041	0.026	0.977	0.0062	0.046
Modified Page	0.993	0.0036	0.041	0.991	0.0025	0.061	0.993	0.0032	0.011
Modified Henderson and pabis	0.987	0.0015	0.010	0.978	0.0042	0.015	0.991	0.0021	0.035
Henderson and Pabis	0.991	0.0062	0.002	0.993	0.0048	0.003	0.982	0.009	0.005
Two-term	0.959	0.0027	0.005	0.949	0.0083	0.004	0.981	0.062	0.022
Thomson	0.923	0.0031	0.031	0.952	0.0021	0.051	0.963	0.0040	0.063
Midilli and Kucuk	0.999	0.0002	0.001	0.997	0.0004	0.0001	0.999	0.0001	0.002
	Air velocity 0.7 m/s								
	1500 W/m^2^			3500 W/m^2^			5500 W/m^2^		
	R^2^	χ^2^	RMSE	R^2^	χ^2^	RMSE	R^2^	χ^2^	RMSE
Logarithmic model	0.993	0.0018	0.051	0.991	0.0059	0.051	0.964	0.0006	0.016
Page	0.990	0.0001	0.027	0.989	0.0020	0.074	0.998	0.0024	0.041
Newton	0.998	0.0005	0.046	0.987	0.0028	0.081	0.998	0.0098	0.061
Wang and Singh	0.984	0.0116	0.034	0.998	0.0033	0.035	0.988	0.0001	0.081
Verma et al.	0.998	0.0008	0.026	0.997	0.0052	0.081	0.998	0.0007	0.012
Modified Page	0.986	0.0009	0.061	0.998	0.0014	0.066	0.998	0.0025	0.052
Modified Henderson and pabis	0.998	0.0025	0.015	0.991	0.0023	0.051	0.998	0.0005	0.019
Henderson and Pabis	0.979	0.0003	0.003	0.995	0.0002	0.004	0.998	0.0084	0.009
Two-term	0.959	0.0146	0.004	0.997	0.0007	0.061	0.940	0.0062	0.008
Thomson	0.995	0.0015	0.051	0.991	0.0026	0.084	0.990	0.0052	0.052
Midilli and Kucuk	0.999	0.0091	0.0001	0.999	0.0051	0.001	0.998	0.0001	0.002
	Air velocity 1 m/s								
	1500 W/m^2^			3500 W/m^2^			5500 W/m^2^		
	R^2^	χ^2^	RMSE	R^2^	χ^2^	RMSE	R^2^	χ^2^	RMSE
Logarithmic model	0.995	0.003	0.065	0.989	0.0023	0.085	0.996	0.003	0.085
Page	0.998	0.0051	0.054	0.995	0.0025	0.045	0.997	0.0051	0.045
Newton	0.995	0.002	0.099	0.993	0.0096	0.036	0.995	0.002	0.036
Wang and Singh	0.995	0.0096	0.036	0.998	0.0036	0.096	0.988	0.0096	0.096
Verma et al.	0.985	0.0025	0.024	0.9998	0.0009	0.036	0.991	0.0025	0.036
Modified Page	0.988	0.0036	0.088	0.995	0.0012	0.014	0.992	0.0036	0.014
Modified Henderson and pabis	0.974	0.0052	0.0087	0.998	0.0009	0.025	0.989	0.0052	0.025
Henderson and Pabis	0.951	0.0041	0.0097	0.995	0.00251	0.088	0.988	0.0041	0.088
Two-term	0.977	0.003	0.081	0.981	0.0085	0.035	0.997	0.0014	0.051
Thomson	0.981	0.015	0.0085	0.994	0.0063	0.081	0.987	0.0052	0.065
Midilli and Kucuk	0.999	0.0085	0.0068	0.999	0.0006	0.008	0.998	0.0085	0.088

**Table 3:** The statistical variables were found by fitting numerous thin-layer drying equations at an air temperature of 50 °C.

Models’ name	Air velocity 0.3 m/s								
	1500 W/m^2^			3500 W/m^2^			5500 W/m^2^		
	*R* ^2^	χ^2^	RMSE	*R* ^2^	χ^2^	RMSE	*R* ^2^	χ^2^	RMSE
Logarithmic model	0.902	0.0017	0.010	0.996	0.0012	0.054	0.996	0.0063	0.031
Page	0.992	0.0031	0.052	0.990	0.0032	0.027	0.981	0.0021	0.067
Newton	0.994	0.0051	0.032	0.992	0.0041	0.046	0.995	0.0031	0.076
Wang and Singh	0.993	0.0023	0.052	0.995	0.0030	0.034	0.991	0.0041	0.014
Verma et al.	0.980	0.0061	0.023	0.975	0.0041	0.026	0.977	0.0062	0.046
Modified Page	0.993	0.0036	0.041	0.991	0.0025	0.061	0.993	0.0032	0.011
Modified Henderson and pabis	0.987	0.0015	0.010	0.978	0.0042	0.015	0.991	0.0021	0.035
Henderson and Pabis	0.995	0.0062	0.002	0.993	0.0048	0.003	0.982	0.009	0.005
Two-term	0.959	0.0027	0.005	0.949	0.0083	0.004	0.983	0.062	0.022
Thomson	0.925	0.0031	0.031	0.951	0.0021	0.051	0.963	0.0040	0.063
Midilli and Kucuk	0.998	0.0002	0.001	0.997	0.0004	0.0001	0.999	0.0001	0.002
	Air velocity 0.7 m/s								
	1500 W/m^2^			3500 W/m^2^			5500 W/m^2^		
	R^2^	χ^2^	RMSE	R^2^	χ^2^	RMSE	R^2^	χ^2^	RMSE
Logarithmic model	0.991	0.0018	0.051	0.991	0.0059	0.051	0.964	0.0006	0.016
Page	0.990	0.0001	0.027	0.989	0.0021	0.074	0.998	0.0024	0.041
Newton	0.999	0.0005	0.046	0.987	0.0028	0.081	0.998	0.0098	0.061
Wang and Singh	0.984	0.0116	0.034	0.998	0.0033	0.035	0.988	0.0001	0.081
Verma et al.	0.998	0.0008	0.026	0.997	0.0052	0.081	0.998	0.0007	0.012
Modified Page	0.986	0.0009	0.061	0.998	0.0015	0.066	0.998	0.0025	0.052
Modified Henderson and pabis	0.998	0.0025	0.015	0.991	0.0023	0.051	0.998	0.0005	0.019
Henderson and Pabis	0.972	0.0003	0.003	0.995	0.0002	0.004	0.998	0.0084	0.009
Two-term	0.959	0.0146	0.004	0.997	0.0007	0.061	0.940	0.0062	0.008
Thomson	0.992	0.0015	0.051	0.991	0.0026	0.084	0.990	0.0052	0.052
Midilli and Kucuk	0.998	0.0091	0.0001	0.999	0.0051	0.001	0.998	0.0001	0.002
	Air velocity 1 m/s								
	1500 W/m^2^			3500 W/m^2^			5500 W/m^2^		
	R^2^	χ^2^	RMSE	R^2^	χ^2^	RMSE	R^2^	χ^2^	RMSE
Logarithmic model	0.995	0.003	0.065	0.989	0.0023	0.085	0.996	0.003	0.085
Page	0.998	0.0051	0.054	0.995	0.0015	0.045	0.997	0.0051	0.045
Newton	0.995	0.002	0.099	0.993	0.0096	0.036	0.995	0.002	0.036
Wang and Singh	0.995	0.0096	0.036	0.998	0.0036	0.096	0.988	0.0096	0.096
Verma et al.	0.981	0.0025	0.024	0.9998	0.0009	0.036	0.991	0.0025	0.036
Modified Page	0.988	0.0036	0.088	0.995	0.0012	0.014	0.992	0.0036	0.014
Modified Henderson and pabis	0.974	0.0052	0.0087	0.998	0.0014	0.025	0.989	0.0052	0.025
Henderson and Pabis	0.951	0.0041	0.0097	0.995	0.00251	0.088	0.988	0.0041	0.088
Two-term	0.978	0.003	0.081	0.981	0.0085	0.035	0.997	0.0014	0.051
Thomson	0.981	0.015	0.0085	0.994	0.0063	0.081	0.987	0.0052	0.065
Midilli and Kucuk	0.998	0.0085	0.0068	0.999	0.0006	0.008	0.998	0.0085	0.088

**Table 4:** The statistical variables were found by fitting numerous thin-layer drying equations at an air temperature of 60 °C.

Models’ name	Air velocity 0.3 m/s								
	1500 W/m^2^			3500 W/m^2^			5500 W/m^2^		
	*R* ^2^	χ^2^	RMSE	*R* ^2^	χ^2^	RMSE	*R* ^2^	χ^2^	RMSE
Logarithmic model	0.852	0.0019	0.010	0.991	0.0017	0.034	0.856	0.0033	0.036
Page	0.992	0.0031	0.052	0.990	0.0032	0.027	0.981	0.0021	0.067
Newton	0.994	0.0051	0.032	0.992	0.0041	0.046	0.995	0.0031	0.076
Wang and Singh	0.993	0.0023	0.052	0.995	0.0030	0.034	0.991	0.0041	0.014
Verma et al.	0.980	0.0061	0.023	0.975	0.0041	0.026	0.977	0.0062	0.046
Modified Page	0.993	0.0036	0.041	0.991	0.0025	0.061	0.993	0.0032	0.011
Modified Henderson and pabis	0.987	0.0015	0.010	0.978	0.0042	0.015	0.991	0.0021	0.035
Henderson and Pabis	0.995	0.0062	0.002	0.993	0.0048	0.003	0.982	0.009	0.005
Two-term	0.959	0.0027	0.005	0.949	0.0083	0.004	0.983	0.062	0.022
Thomson	0.925	0.0031	0.031	0.951	0.0021	0.051	0.963	0.0040	0.063
Midilli and Kucuk	0.998	0.0002	0.001	0.997	0.0004	0.0001	0.999	0.0001	0.002
	Air velocity 0.7 m/s								
	1500 W/m^2^			3500 W/m^2^			5500 W/m^2^		
	R^2^	χ^2^	RMSE	R^2^	χ^2^	RMSE	R^2^	χ^2^	RMSE
Logarithmic model	0.991	0.0018	0.051	0.991	0.0059	0.051	0.964	0.0006	0.016
Page	0.990	0.0001	0.027	0.989	0.0020	0.074	0.998	0.0024	0.041
Newton	0.999	0.0005	0.046	0.987	0.0028	0.081	0.998	0.0098	0.061
Wang and Singh	0.984	0.0116	0.034	0.998	0.0033	0.035	0.988	0.0001	0.081
Verma et al.	0.998	0.0008	0.026	0.997	0.0052	0.081	0.998	0.0007	0.012
Modified Page	0.986	0.0009	0.061	0.998	0.0014	0.066	0.998	0.0025	0.052
Modified Henderson and pabis	0.998	0.0025	0.015	0.991	0.0023	0.051	0.998	0.0005	0.019
Henderson and Pabis	0.972	0.0003	0.003	0.995	0.0002	0.004	0.998	0.0084	0.009
Two-term	0.959	0.0146	0.004	0.997	0.0007	0.061	0.940	0.0062	0.008
Thomson	0.992	0.0015	0.051	0.991	0.0026	0.084	0.990	0.0052	0.052
Midilli and Kucuk	0.998	0.0091	0.0001	0.999	0.0051	0.001	0.998	0.0001	0.002
	Air velocity 1 m/s								
	1500 W/m^2^			3500 W/m^2^			5500 W/m^2^		
	R^2^	χ^2^	RMSE	R^2^	χ^2^	RMSE	R^2^	χ^2^	RMSE
Logarithmic model	0.995	0.003	0.065	0.989	0.0023	0.085	0.996	0.003	0.085
Page	0.998	0.0051	0.054	0.995	0.0025	0.045	0.997	0.0051	0.045
Newton	0.995	0.002	0.099	0.993	0.0096	0.036	0.995	0.002	0.036
Wang and Singh	0.995	0.0096	0.036	0.998	0.0036	0.096	0.988	0.0096	0.096
Verma et al.	0.981	0.0025	0.024	0.9998	0.0009	0.036	0.991	0.0025	0.036
Modified Page	0.988	0.0036	0.088	0.995	0.0012	0.014	0.992	0.0036	0.014
Modified Henderson and pabis	0.974	0.0052	0.0087	0.998	0.0009	0.025	0.989	0.0052	0.025
Henderson and Pabis	0.951	0.0041	0.0097	0.995	0.00251	0.088	0.988	0.0041	0.088
Two-term	0.978	0.003	0.081	0.981	0.0085	0.035	0.997	0.0014	0.051
Thomson	0.981	0.015	0.0085	0.994	0.0063	0.081	0.987	0.0052	0.065
Midilli and Kucuk	0.998	0.0085	0.0068	0.999	0.0006	0.008	0.998	0.0085	0.088

The Midilli and Kucuk model is a drying model that considers various factors such as the initial moisture content, drying rate, and equilibrium moisture content. It has a more complex structure compared to some simple drying models. For example, it may have additional terms that account for the drying process’s non-linear behavior. It is designed to handle the changing drying rate over time. For instance, the model can still accurately predict the drying behavior when the material approaches its equilibrium moisture content in the later drying stages. This is a more realistic assumption. In many drying processes, moisture movement from the material’s interior to its surface and the evaporation of moisture from the surface to the surrounding air play essential roles^1^. In contrast, some simpler models might assume that the drying rate is only controlled by a single factor, such as the drying conditions. These simpler models may not accurately capture the drying process’s complexity. Some other models may have fewer parameters, limiting their ability to capture the nuances of the data. A model with only one or two parameters may not be able to adjust to the complex relationships present in the drying data or the Midilli and Kucuk models^55^. The (k) increases with higher infrared levels and decreases with drying airflow. The coefficients of a, n, b, and constant k (min^− 1^) were utilized in a multiple regression versus radiation (I) and drying air velocity (V) in m/s to account for the effect of drying variables on the Midilli and Kucuk model. The equations expressing these correlations take the form of Eqs. 30–33.

30 $$k=0.018+0.12 \left(I\right)-0.051 \left(V\right)-0.30 {(I}^{2})+0.014 {(V}^{2})+0.029 (I.V) {R}^{2}=0.919$$

31 $$a=2.0-2.98 \left(I\right)+0.42 \left(V\right)+7.10 {(I}^{2})-0.013 {(V}^{2})+0.024 (I.V) {R}^{2}=0.911$$

32 $$n=1.74-0.40 \left(I\right)+0.09 \left(V\right)+0.98 {(I}^{2})-0.057 ({V}^{2})-0.21 (I.V ) {R}^{2}=0.822$$

33 $$b=0.0020-0.04 \left(I\right)+0.0069 \left(V\right)+0.21 \left({I}^{2}\right)-0.007 {(V}^{2})-0.0019 (I.V) {R}^{2}=0.80$$

The Midilli and Kucuk model best fits the experimental drying data, with the highest R^2^ and lowest χ^2^ and RMSE values among these seven mathematical models. Since the data were on the 45° line, the predicted values agreed with the experimental drying values. The Midilli and Kucuk effectively represent the dehydrating performance of onion under various airflow and power conditions^56^.

### Effective moisture diffusivity and activation energy

Mass transfer by diffusion is the movement of water or other volatile substances from the material’s interior to the surface due to a concentration gradient. Fick’s first law of diffusion describes the rate of diffusion. In drying, the water concentration is higher in the interior of the wet material and lower at the surface exposed to the drying medium. The diffusion coefficient depends on factors such as the nature of the material, temperature, and moisture content. For example, in a porous material like a sponge, water diffuses through the pores from the regions of high water to the areas of low water. As the drying process progresses, the diffusion path length for water molecules increases as the outer layers of the material dry out, and the diffusion rate may decrease^57^.

The saturation vapor pressure is a function of temperature. As the material’s surface temperature increases, the saturation vapor pressure also increases, enhancing the evaporation rate. In addition, reducing the partial pressure of water vapor in the drying air also accelerates the evaporation process. In most drying processes, heat and mass transfer co-occur. Heat transfer provides the energy required for water evaporation and water molecules’ movement through the material. In contrast, mass transfer removes the water vapor from the drying environment, allowing the drying process to continue. The efficiency of the drying process depends on the proper balance and optimization of these heat and mass transfer mechanisms^58^. Due to the volumetric heating effect in infrared drying, moisture in the material’s interior can vaporize more rapidly. The increased temperature in the interior creates a strong moisture gradient within the material. This enhanced gradient can accelerate moisture diffusion from the interior to the surface. In convection drying, moisture diffusion is mainly influenced by the surface conditions. As the surface dries, the moisture concentration decreases, creating a concentration gradient from the interior to the surface. Moisture diffuses from the regions of higher concentration to those of lower concentration. However, because the heating is surface-driven, the surface may dry too quickly in some cases, reducing the moisture gradient and slowing down the diffusion from the interior^59^.

**Fig. 2:**
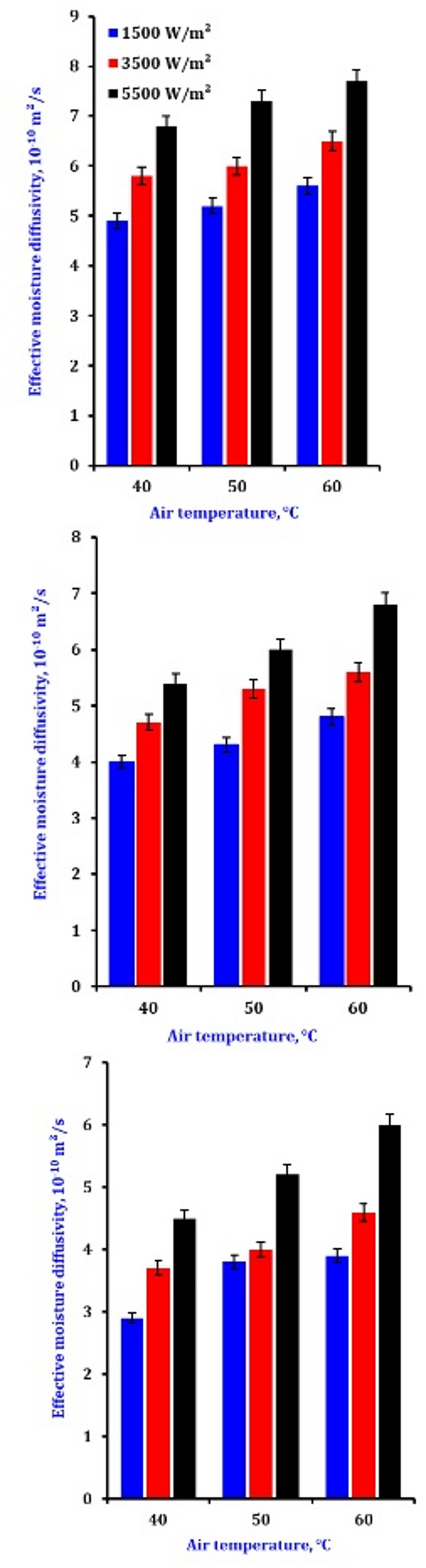
The effect of drying conditions on the effective moisture diffusivity of onion slices.

Figure 2 shows the effective moisture diffusivity calculated using Fick’s second diffusion rule. The *D*_*eff*_ was estimated by operating the slope method. The optimum moisture diffusivity results for drying slices are 5.05, 6.11, and 3.65 10^−10^ at 1500, 3500, and 5500 W/m² under 0.3 m/s, respectively. The results showed that moisture diffusivity, rising slice thickness, and infrared intensity improved. The average energy for a vapor’s transitional, rotational, and vibrational motions increases with increasing intensity, causing a more significant moisture gradient, a higher mass transfer rate, and increased moisture diffusivity. The optimum moisture diffusivity decreased with the airflow going from 0.3 to 1.0 m/s. This could be because the higher airflow reduced the samples’ temperatures, as their temperature was higher than the airflow. The (Ea) ranged between 18 and 28.95 kJ/mol under various experimental conditions, within the acceptable activation energy range for most goods (12.7–110 kJ/mol)^60^. At every level of infrared, activation energy values rose as airflow surged between 0.5 and 1.5 m/s. During infrared drying, the dehydrating rate dropped as the airflow grew between 0.3 and 1.0 m/s. The measurements of (Ea) of a process drop as the mean energy of the molecules increases^61^.

### Water activity

**Fig. 3:**
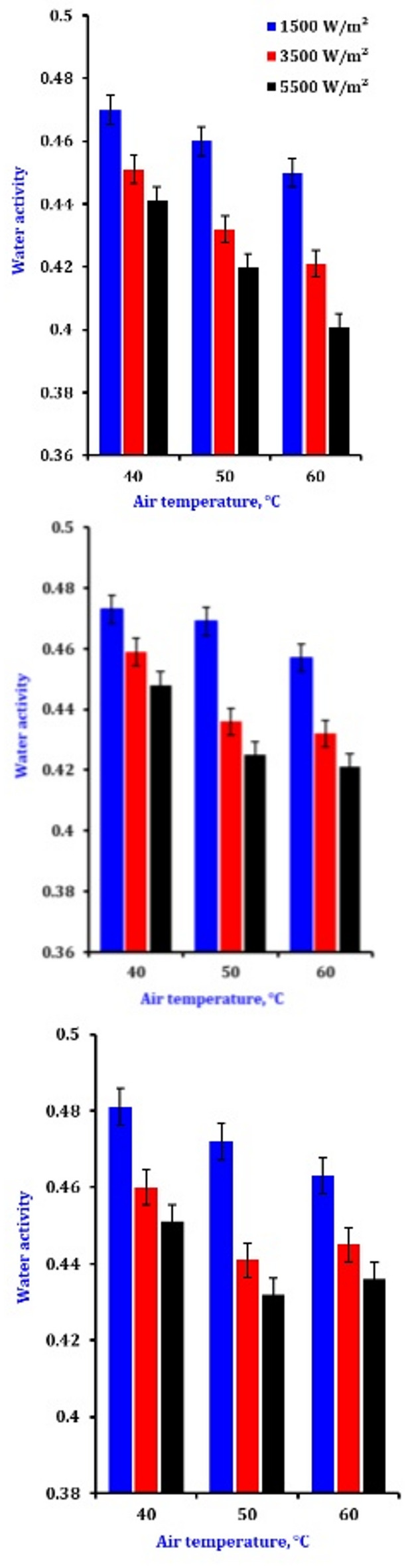
The effect of drying conditions on the water activity of onion slices.

Figure 3 displays the impact of different drying settings on the (aw) of the samples. According to all drying requirements, the (a_w_) slices remain constantly below 0.6, indicating that the samples are not deteriorating due to microbial activity (Table 5). The increased infrared and reduced airflow cause water to disappear from the exteriors of the onion. The improved water loss from the superficial slice and increased water diffusion within the slices facilitate more rapid attainment of the desired water activity^62^. Speedier achievement of the required (a_w_) is made possible by more forceful water loss from the slice and more active diffusion of moisture inside the slices^62,63^. The results of our study demonstrate that infrared drying techniques can effectively attain the desired water activity appropriate for prolonged storage durations.

### Colour attributes

**Fig. 4:**
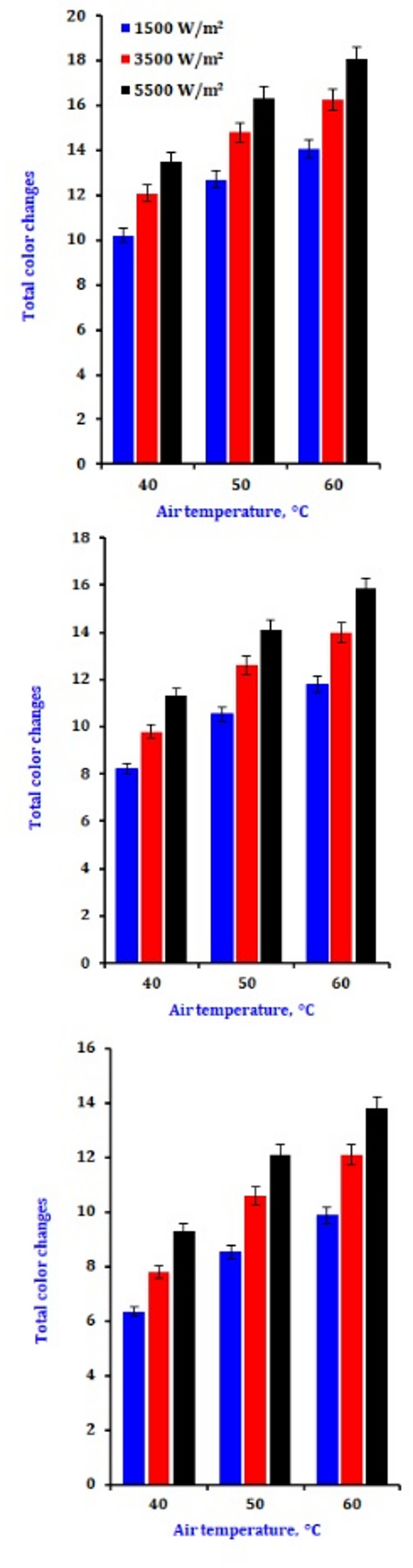
The influence of drying conditions on the total color changes of dry onion slices.

Figure 4 shows the outcome of drying settings on total color changes. The color variations rose when the airflow was 0.3 m/s, and the IR ranged from 1500 to 5500 W/m². The same radiation power range was applied, and a fixed drying air velocity of 1.0 m/s increased color variation (Table 5). The colour change is accompanied by increased airflow and IR^64^. The total color difference increased with drying air temperature and infrared intensity. This can be attributed to pods being exposed to heat during drying, which caused enzymatic and nonenzymatic browning reactions, which are potentiated by higher temperature and air velocity drying conditions^65^.

This finding aligns with^66^who found that extended dehydration processes lead to more pronounced browning reactions. These reactions affect the compounds in the samples and likely contribute to pigment degradation as well as enzymatic and non-enzymatic reactions. The δE increased with higher infrared intensity but decreased with greater airflow.

The total color change of the onion amplified with an increase in infrared intensity at a constant drying airflow. Still, it decreased by drying airflow at a continuous infrared radiation intensity^67^. The decrease in colour difference with air velocity might be credited to cooling the slice’s surface with flowing air within the drying chamber, producing an entire heat loss at the drying chamber^68^. Tanta and Doymaz^69^ found that high infrared intensity increases the color difference of dried okra values at infrared drying. Taghinezhad et al.^70^ investigated the overall color change in organic blackberries during hybrid hot air/infrared drying, both with and without ultrasonic pretreatment, for 15, 30, and 45 min. Their results showed a consistent decrease in the total color variation of the samples as the drying air temperature increased from 50 to 60 °C and from 60 to 70 °C.

### Browning index

**Fig. 5:**
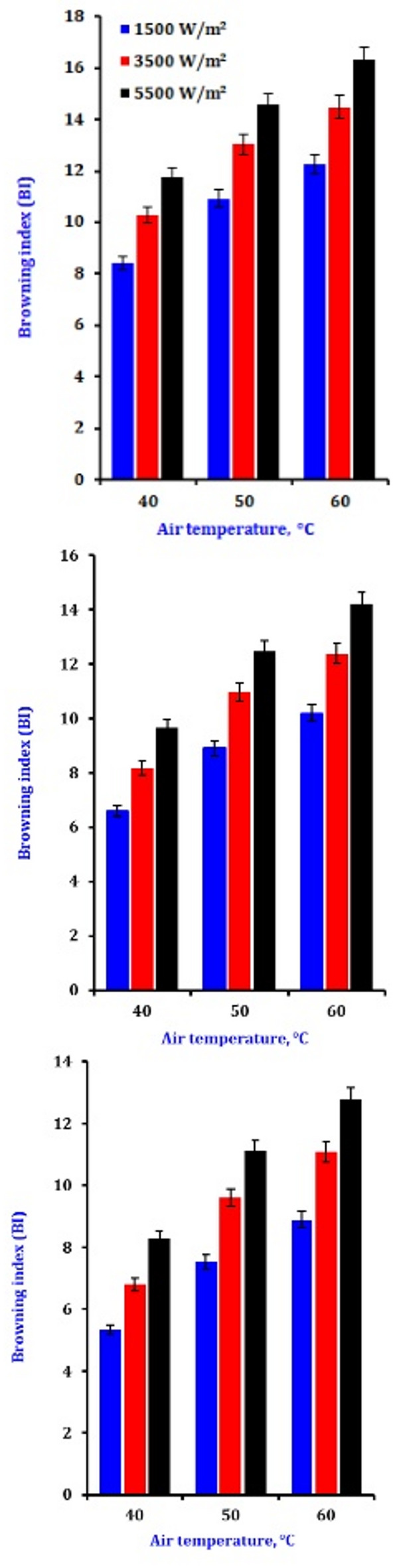
The impact of drying conditions on the browning index of dry onion slices.

Non-enzymatic browning serves as an additional quality indicator during the drying process. Browning is advantageous in certain processed foods but unfavorable in dried onion. The degree of browning is primarily due to color changes induced by Maillard reactions in onions. The browning index (BI) values for dried onion are illustrated in Fig. 5; Table 5. Infrared drying exhibited a lower BI value in the dried samples than in the fresh sample, attributed to pigment decomposition occurring during prolonged drying^71^. Conversely, reduced L values were noted in other dried samples, resulting in elevated BI values. The findings indicate that integrating infrared and microwave drying of blueberries facilitated the browning reaction, producing a product with a darker hue. The brightness index (BI) rises with an increase in infrared power. This trend was similarly noted in the dried onion slices. Onion samples were subjected to drying in an aerobic environment using infrared methods. The elevated sample temperature enhanced the Maillard reaction and ascorbic acid oxidation, forming brown-colored substances^72^. At an infrared power level of 5500 W/m^2^, an increase in airflow resulted in a higher BI value. The elevated temperature of the infrared power facilitated the browning reaction and starch gelatinization. The BI value of the dried sample was influenced by both the sample temperature and the drying duration.

### Shrinkage ratio

**Fig. 6:**
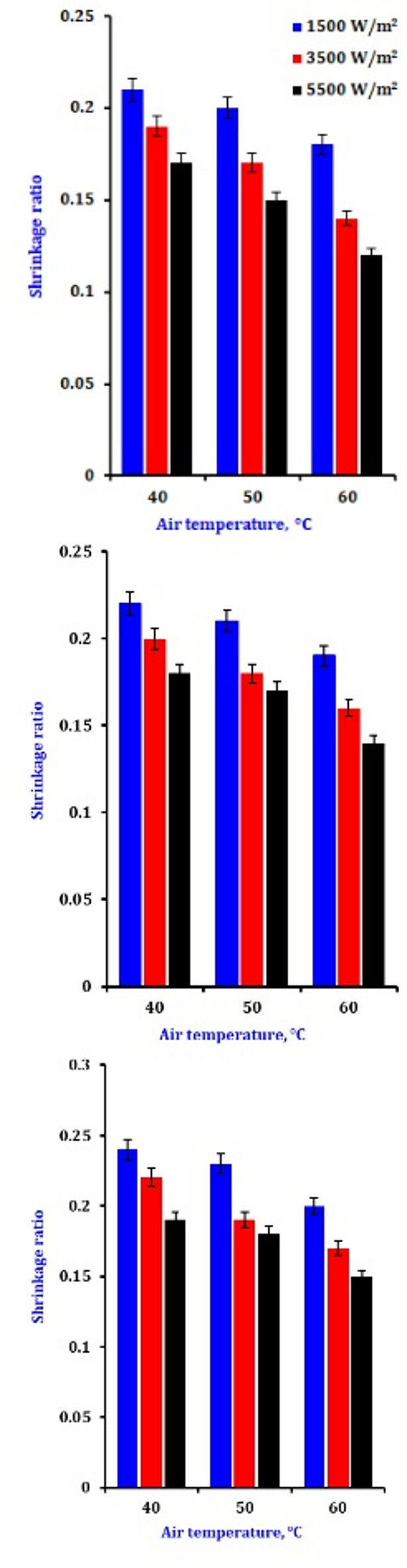
The impression of drying conditions on the shrinkage ratio of dry onion slices.

The (Sr) samples dehydrated through infrared are shown in Fig. 6. With the power increased from 1500 to 5500 W/m², the (Sr) released decreased from 0.25 to 0.16, and the airflow was maintained at 0.3 m/s. The Sr decreased from 0.25 to 0.21 while maintaining the same IR power and airflow of 1.0 m/s (Table 5). Therefore, the lowest values of the shrinkage ratio were obtained by operating at a low airflow and a considerable infrared radiation intensity of 1500 W/m². Under increasing intensity and low airflow conditions, a small amount of shrinkage occurs on both sides of the onion’s outer surfaces. This is because the product temperatures are more significant, and the moisture content is lower^73^. Foodstuff shrinkage is a regular physical occurrence seen throughout various drying techniques. It affects the quality of the dehydrated products. It should be considered when predicting moisture and temperature profiles in the dried material. Feng et al.^27^ reported a trend in the air drying of garlic at different process temperatures,, but with various degrees of shrinkage due to the different drying conditions used. It was also found that a lower dehydration capacity corresponded to a sample that sustained higher shrinkage effects. This reduction is consistent with the typical shrinkage observed in samples due to dehydration during drying. Removing moisture from tissues creates a pressure differential between the tissue’s interior and outside, which leads to compressive stresses and shrinking.

### Rehydration ratio

**Fig. 7:**
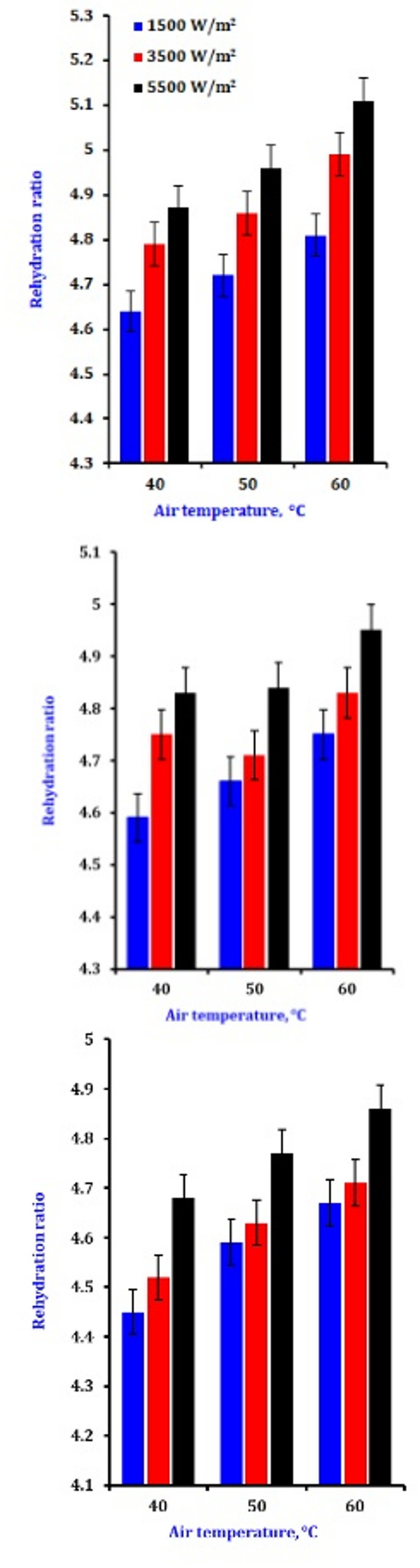
The impression of drying conditions on the rehydration ratio of dry onion slices.

The ratio of rehydration is a critical quality attribute for dried food materials. Rehydration is a complex process influenced by physical and chemical changes associated with drying and the treatments preceding dehydration. The rehydration ratio is shown after the slices have been dried using various drying air velocities and infrared radiation intensities (Fig. 7). The dryer reaches its lowest value of 3.5 when operating at the lightest airflow and the greatest IR, and it reaches its highest value of 5.1 when operating at the shallowest airflow and the highest IR. With an increase in the IR, it is evident that the rehydration ratio drops as the airflow increases. Regarding changes in the rehydration ratio, increasing or decreasing the air velocity has a higher influence than changing the radiation intensity (Table 5). It is possible to argue that the objects heated up more rapidly when exposed to greater infrared intensities, accelerating the vapor generation inside the object and increasing its porosity^74^. also reported that IR improved rehydration by reducing the drying time. Consequently, IR exposure on the slice surfaces created mini-channels, which later became pores, facilitating more efficient water transmission to the samples. Additionally, the rehydration ratio increased with rising air temperatures. This suggests that the air temperature may cause variations in the product, potentially leading to a loss of solids during rehydration, thereby facilitating rehydration. This effect arises from the increased IR, which raises the air velocity around the slices. Consequently, there is an increase in the temperature gradient over the underlying slices or surface layer, accelerating the rate of moisture evaporation. Additionally, the rehydration rate of okra improves with higher drying temperatures. At higher temperatures, the food’s rehydration rate increases because of the temperature’s effect on tissue collapse and cell damage.

### Vitamin C

**Fig. 8:**
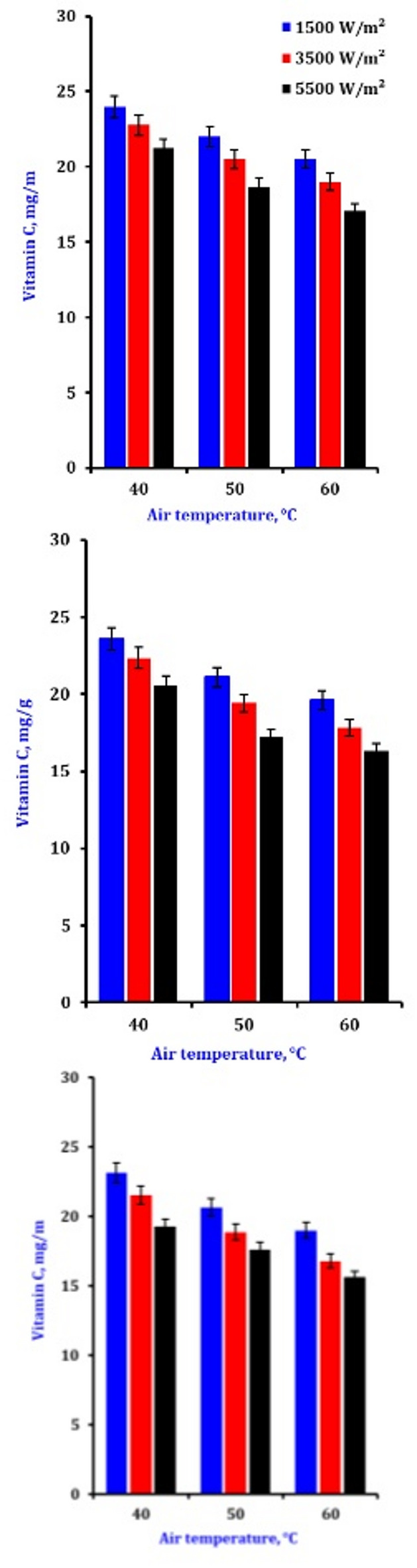
Effect of drying conditions on the vitamin C of onion slices.

The vitamin C found in fresh produce is abundant but readily destroyed by processing methods that expose it to oxygen, heat, or light, making it very unstable. Figure 8 summarizes the effects of different airflows, temperatures, and IR intensities on the vitamin C content of dried onion slices. According to the results, Vitamin C levels drop with rising air temperature and radiation intensity (Table 5). Its quick breakdown due to heat sensitivity is why a drop in vitamin C. Vitamin C can be found in most fresh fruits and vegetables; however, it is not very stable and is usually degraded during processing when subjected to heat, oxygen, or light. The improvement of the conditions for drying garlic using a microwave was examined by Sharma and Prasad^75^. Their hybrid dryer research discovered that extending the airflow from 1.0 to 2.0 m/s while maintaining the same air temperature and infrared intensity level lengthened the dehydrating time. In contrast to what might be anticipated during hot air drying, the drying time increased rather than decreased. One possible explanation is that the material cooled due to the increased air velocity, reducing the slices’ temperature. Nevertheless, with an airflow of 2.0 m/s, 40 W, and 60 °C, the dried sample showed the maximum vitamin C during the drying procedure. Shorter drying times and lower air temperatures best preserve vitamin C while maximizing drying efficiency. Longer drying durations or exposure to higher temperatures and IR intensities accelerate degradation. This is probably because the chemical composition of the onion slices changes, and thermal breakdown occurs due to greater thermal exposure.

### Machine learning modeling

Artificial Neural Networks (ANNs) are robust, data-driven artificial intelligence tools that model complex relationships. An ANN typically comprises three layers: input, hidden, and output. Data flows through the neurons, starting from the input layer and progressing to other layers, where the input and output neurons substantially impact the variables.

Various architectures were evaluated to identify the most efficient ANN model by adjusting the number of hidden layers, neurons in each layer, activation functions, and optimization algorithms. The process of optimization encompasses:

Choosing an Architectural Framework.

- Comparison of Single-layer and Multi-layer Perceptrons (MLP).
- Adjusting the quantity of concealed layers (ranging from 1 to 3 layers).
- Evaluating various neuron quantities for each layer (5, 10, 15, and 20).

Activation Functions.

- The Rectified Linear Unit (ReLU) was employed for the hidden layers because it effectively mitigated the vanishing gradient problem.
- The Sigmoid and Tanh functions were evaluated but demonstrated slower convergence rates.

Optimizing Algorithm.

The Adam optimizer was selected instead of Stochastic Gradient Descent (SGD) because of its superior convergence speed and adaptive learning rate capabilities.

Hyperparameter Tuning.

- Various learning rates (0.001, 0.005, 0.01) were evaluated, with 0.01 demonstrating the most effective balance between speed and accuracy.
- Dropout rates of 0.2, 0.3, and 0.5 were utilized to mitigate overfitting.

Assessment and Evaluation Criteria.

- A 5-fold cross-validation was performed to evaluate the model’s generalization.
- Mean Squared Error (MSE), Root Mean Squared Error (RMSE), and R² were utilized to assess the model’s performance.

The finalized optimized ANN model featured two hidden layers, each comprising 15 neurons, utilized ReLU activation, and employed the Adam optimizer, demonstrating superior performance compared to alternative architectures in predicting onion quality parameters. In many different configurations, the Artificial Neural Network (ANN) is the ideal method for predicting the results of nonlinear structures. Estimating the output data of the intended collection of parameter combinations, overlaying the technique behavior, and filling the network with empirical data are the key components of the procedure. ANN models are combined using extra algorithms to improve dryer settings or monitor the drying process through the self-organizing map (SOM) method.

**Table 5:** Influence of drying conditions on physicochemical properties of dried onion slices using the hybrid infrared-convective heating system.

Infrared intensity, W/m^2^	Air velocity m/s										
	0.3					0.7			1.0		
	Air temperature, °C										
	40			50	60	40	50	60	40	50	60
	Water activity										
1500	0.47 ± 0.63^b^			0.45 ± 0.75^g^	0.44 ± 0.81^ij^	0.47 ± 0.65^b^	0.44 ± 0.58^c^	0.48 ± 0.79^g^	0.46 ± 0.62^a^	0.45 ± 0.77^b^	0.44 ± 0.93^p^
3500	0.47 ± 0.51^i^			0.43 ± 0.63^m^	0.42 ± 0.70^q^	0.46 ± 0.58^f^	0.42 ± 0.69^h^	0.47 ± 0.96^m^	0.44 ± 0.58^d^	0.43 ± 0.99^f^	0.43 ± 0.52^j^
5500	0.45 ± 0.63^l^			0.42 ± 0.62^p^	0.40 ± 0.82^s^	0.45 ± 1.00^k^	0.41 ± 0.87^m^	0.47 ± 0.74^r^	0.43 ± 0.98^h^	0.41 ± 0.68^j^	0.42 ± 0.84^e^
	Clour										
1500	11.21 ± 2.11^o^			12.08 ± 1.87^k^	13.80 ± 1.04^h^	8.74 ± 1.42^r^	9.85 ± 1.41^m^	11.25 ± 1.20^l^	6.25 ± 1.63^s^	7.85 ± 1.05^q^	9.57 ± 1.032^p^
2000	13.08 ± 1.57^h^			14.92 ± 1.86^d^	16.41 ± 1.84^c^	10.14 ± 1.70^n^	12.63 ± 1.55^h^	15.00 ± 1.32^f^	8.88 ± 1.11^r^	10.36 ± 0.98^mn^	12.11 ± 1.02^j^
3000	14.11 ± 1.90^g^			16.87 ± 1.87^b^	18.17 ± 1.93^a^	11.92 ± 0.13^k^	13.52 ± 1.28 ^f^	15.82 ± 1.18^c^	9.87 ± 1.52^o^	12.08 ± 1.07^i^	13.51 ± 1.60^e^
	Browning index										
1500	8.45 ± 0.23 h			10.29 ± 0.22n	11.742 ± 0.22q	6.59 ± 0.2cq	8.65 ± 0.23i	9.66 ± 0.22 lm	5.34 ± 0.22a	6.98 ± 0.24d	8.36 ± 0.23f
2000	10.92 ± 0.22mn			130.2 ± 0.22qn	14.55 ± 0.21r	8.95 ± 0.19f	10.98 ± 0.23k	12.47 ± 0.22op	7.54 ± 0.21b	9.61 ± 0.23nh	11.52 ± 0.22kl
3000	12.28 ± 0.22os			14.47 ± 0.20s	16.29 ± 0.22t	10.18 ± 0.24j	12.37 ± 0.23p	14.21 ± 0.22r	8.95 ± 0.25e	10.08 ± 0.23n	12.96 ± 0.22q
	Shrinkage ratio										
1500	0.21 ± 0.23^g^			0.19 ± 0.22^n^	0.17 ± 0.22^q^	0.22 ± 0.2^c^	0.2 ± 0.23^i^	0.18 ± 0.22^lm^	0.24 ± 0.22^a^	0.22 ± 0.24^d^	0.19 ± 0.23^f^
2000	0.19 ± 0.22^mn^			0.17 ± 0.22^q^	0.15 ± 0.21^r^	0.21 ± 0.19^f^	0.19 ± 0.23^k^	0.17 ± 0.22^op^	0.23 ± 0.21^b^	0.19 ± 0.23^h^	0.18 ± 0.22^kl^
3000	0.18 ± 0.22^o^			0.14 ± 0.20^s^	0.12 ± 0.22^t^	0.19 ± 0.24^j^	0.16 ± 0.23^p^	0.14 ± 0.22^r^	0.21 ± 0.25^e^	0.17 ± 0.23^n^	0.15 ± 0.22^q^
	Rehydration ratio										
1500	4.67 ± 1.08^p^			4.75 ± 1.22^l^	4.87 ± 1.09^g^	4.59 ± 0.09^r^	4.75 ± 1.09^o^	4.75 ± 1.30^j^	4.55 ± 1.36^t^	4.79 ± 1.18^r^	4.67 ± 1.04^n^
2000	4.72 ± 1.19^h^			4.71 ± 1.32^e^	4.99 ± 1.41^b^	4.66 ± 0.11^j^	4.61 ± 1.27^k^	4.84 ± 1.53^f^	4.62 ± 1.32^s^	4.40 ± 1.30^q^	4.71 ± 1.06^k^
3000	4.81 ± 1.0^e^			4.83 ± 1.08^c^	5.11 ± 0.89^a^	4.75 ± 0.08^f^	4.84 ± 1.17^f^	4.81 ± 1.62^d^	4.78 ± 1.07^m^	4.51 ± 1.19^i^	4.86 ± 1.88^e^
	Vitamin C, mg/g										
1500	22.55 ± 1.25^e^			22.81 ± 1.75^g^	21.22 ± 1.50^j^	23.21 ± 1.00^b^	22.35 ± 2.00^e^	20.56 ± 1.50^h^	23.11 ± 1.50^a^	21.52 ± 2.50^c^	19.25 ± 1.00^d^
2000	22.08 ± 1.75^g^			20.52 ± 1.25^i^	18.71 ± 1.00^k^	21.11 ± 1.25^d^	19.43 ± 1.00^f^	17.21 ± 1.25^j^	20.33 ± 1.50^c^	18.8 ± 1.25^d^	17.62 ± 1.75^g^
3000	20.55 ± 1.50^j^			19.08 ± 1.00^k^	17.05 ± 1.50^m^	19.63 ± 2.25^h^	17.81 ± 2.25^i^	16.34 ± 1.00^l^	18.98 ± 2.75^e^	16.78 ± 1.75^g^	15.62 ± 1.00^k^

The variable’s values are average ± SD. The column values with the same letters are statistically similar according to the Duncan Multiple Range Test (DMRT) at *p* < 0.05.

This study selected Artificial Neural Networks (ANNs) for their robust ability to model intricate, non-linear relationships between drying conditions and onion quality parameters. In contrast to conventional regression models, ANNs can discern complex patterns within extensive datasets, rendering them effective for forecasting physicochemical and enzymatic alterations in onion as time progresses. Additionally, the ANN model exhibited enhanced prediction accuracy, characterized by reduced Mean Squared Error (MSE) and elevated R² values. The capacity of ANN to grasp multi-dimensional interactions among input variables played a significant role in its designation as the top-performing model. Furthermore, when provided with an adequate dataset, ANNs demonstrate strong generalization capabilities, improving their predictive accuracy for upcoming storage scenarios.

Concurrently, the sigmoid function, represented by Eq. (40), is applied across all instances. The sigmoid function maps input values to an output range between 0 and 1, introducing nonlinearity into the model, which is essential for learning complex patterns.

40 $$\sigma \left(x\right)=\frac{1}{1+{e}^{-x}}$$

Data input proceeds from the input layer through the neurons to subsequent layers, with the dynamics contingent on the neurons in both the input and output layers. The algorithm undergoes four phases: initialization, activation, weight adjustment, and iterative training to refine the dataset. Before integration into the ANN structure, the data is normalized as per Eq. (41), ensuring that the values range between − 1.0 and 1.0:

41 $${X}_{i}=\frac{{x}_{i,max}-{x}_{i}}{{x}_{i,max}-{x}_{i,min}}$$

Error minimization is achieved through the Levenberg-Marquardt algorithm, with the least error observed at 1000 iterations. The Root Mean Square Error (RMSE), outlined in Eq. (42), serves as the metric for modeling accuracy, with a value approaching zero indicating optimal precision. Here, ${X}_{m}$ denotes the measured data, and ${X}_{p}$represents the predicted values:

42 $$RMSE=\sqrt{\frac{1}{n}\sum _{i=1}^{n}{\left({X}_{m}-{X}_{p}\right)}^{2}}$$

**Fig. 9:**
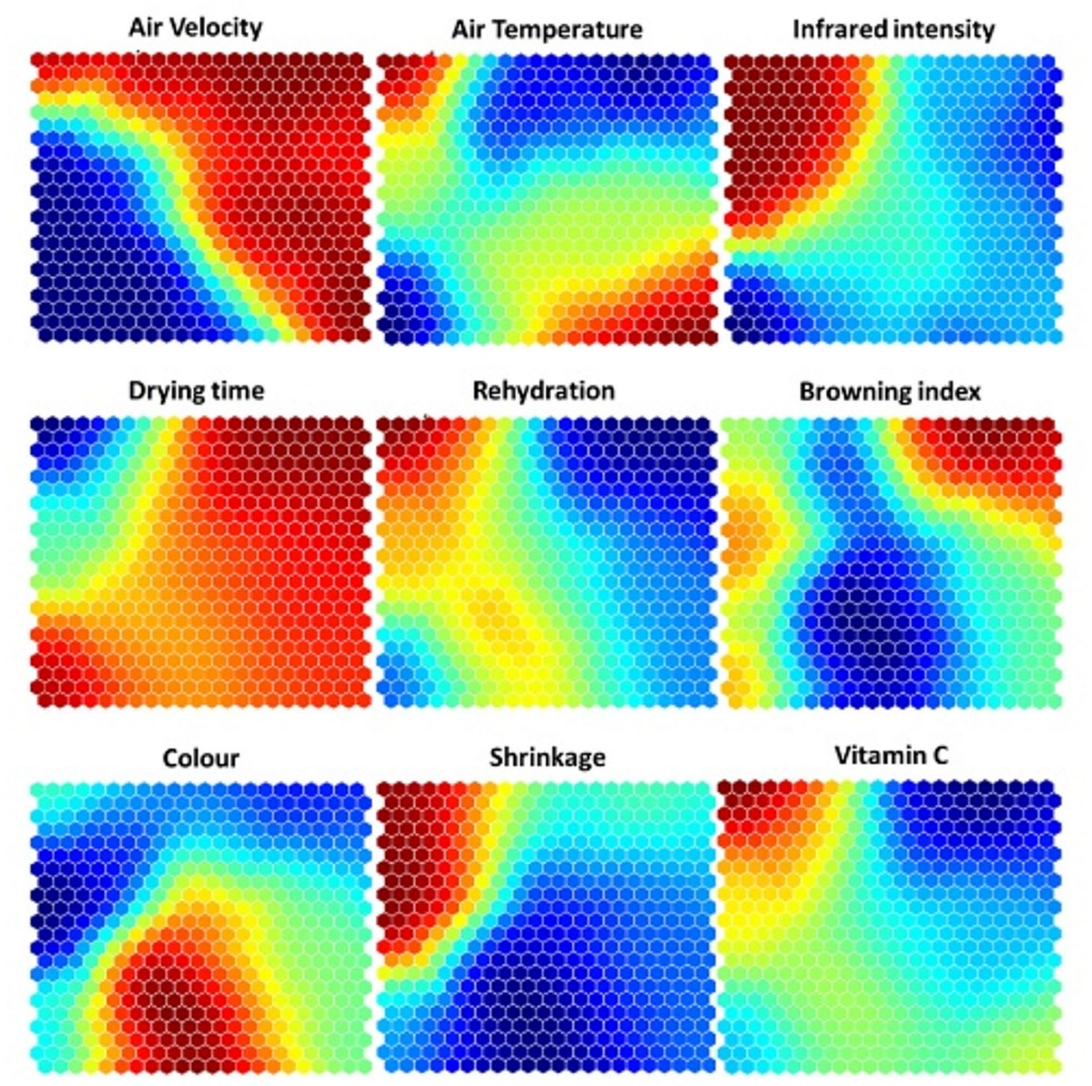
Self-organizing maps of input and output variables of drying onion slices.

Although the previous evaluation gives an extensive overview of the quality material, self-organizing maps (SOM), an exciting information extraction approach, can provide an alternative knowledge of the best working conditions. The SOM representations are shown in Fig. 9, which are grouped into five clusters according to feature similarity. While this process produces appropriate levels of allicin, it is more exhausting and does not improve quality parameters. The map shows low rehydration, high shrinkage, and low color in the final cluster. Thus, onion drying with high IR, low T, and V is guided. The colour indicates relatively low-to-medium values, while the vitamin C shows medium-to-high values. This cluster has a wide range of convection and radiation changes, from low to high values. The second cluster’s water activity exhibits lower values, while the rehydration and color show medium values.

## Conclusions

This study investigated the influence of slice thickness, airflow, infrared power drying behavior, and the physico-chemical quality parameters of onions. The drying period rises with rising airflow, independent of IR. The findings demonstrate that water activity generated by the IR drying process exhibits adequate stability for storage. The experiment’s findings indicate that the model proposed by “Midilli and Kucuk” most effectively elucidates the drying process of onion slices in a thin layer. In the experimental infrared intensity range of 1500–5500 W/m^2^, the effective moisture diffusivity values varied across different experimental conditions, from 2.6 to 8.9 × 10^−10^ m^2^/s. With a decrease in airflow and an increase in the rehydration ratio, there was a corresponding rise in thickness and infrared power. With an increase in airflow, the Sr exhibited an upward trend; conversely, an increase in slice thickness and infrared intensity resulted in a decline. The colour difference increased with the intensity of the infrared light. The degree of browning is primarily due to color changes induced by Maillard reactions in onions. The brightness index (BI) rises with an increase in infrared power. Controlling infrared power and airflow enhances drying qualities and browning efficiency.

## Data Availability

The original contributions presented in the study are included in the article; further inquiries can be directed to the author (Hany S. El-Mesery, elmesiry@ujs.edu.cn) and the corresponding author.
